# Obtention of Sacha Inchi (*Plukenetia volubilis Linneo*) Seed Oil Microcapsules as a Strategy for the Valorization of Amazonian Fruits: Physicochemical, Morphological, and Controlled Release Characterization

**DOI:** 10.3390/foods11243950

**Published:** 2022-12-07

**Authors:** Aureliano Rodríguez-Cortina, Jader Rodríguez-Cortina, María Hernández-Carrión

**Affiliations:** 1 Grupo de Diseño de Productos y Procesos (GDPP), Department of Chemical and Food Engineering, Universidad de los Andes, Bogotá 111711, Colombia; 2 Centro de Investigación Tibaitatá, Corporación Colombiana de Investigación Agropecuaria—Agrosavia, Mosquera 250047, Colombia

**Keywords:** controlled oil release, emulsion, micro-encapsulation, omega-3, bioavailability, sodium caseinate

## Abstract

Sacha inchi seed oil (SIO) is a promising ingredient for the development of functional foods due to its large amount of high-value compounds; however, it is prone to oxidation. This work aimed to obtain SIO microcapsules using conventional and ultrasound probe homogenization and using spray- and freeze-drying technologies as effective approaches to improve the long-term stability of functional compounds. The application of ultrasound probe homogenization improved the rheological and emulsifying properties and decreased the droplet size and interfacial tension of emulsions. The microcapsules obtained by both drying technologies had low moisture (1.64–1.76) and water activity (0.03–0.11) values. Spray-dried microcapsules showed higher encapsulation efficiency (69.90–70.18%) compared to freeze-dried ones (60.02–60.16%). Thermogravimetric analysis indicated that heat protection was assured, enhancing the shelf-life. Results suggest that both drying technologies are considered effective tools to produce stable microcapsules. However, spray-drying technology is positioned as a more economical alternative to freeze-drying.

## 1. Introduction

Research, development, and commercialization of functional food ingredients have attracted significantly more attention from the scientific community and the food industry in the last decades [1]. Nowadays, the addition of bioactive compounds is one of the most widely used methods for the elaboration of functional foods [2]. Compounds such as proteins, fatty acids, polyphenols, carotenoids, vitamins, and minerals, among others, have been investigated for their potential applications in food product fields [3]. It is increasingly common to find foods rich in polyunsaturated fatty acids (PUFA) [4,5]. This is due to the many benefits that these compounds provide for the prevention of cardiovascular diseases and immune response disorders [6].

Extraction of some natural fats or oils,—like flaxseed and chia oil, or marine animal oils, such as salmon, tuna, and sardine, among others—can be used to obtain many fatty acids, especially polyunsaturated ones, which are difficult to synthesize [7]. Sacha inchi (*Plukenetia volubilis Linneo*) is a species native to the Peruvian Amazon [7]. It has recently been considered a promising species because it easily adapts to cropland and its seeds contain an oil rich in omega-3, 6, and 9 fatty acids and vitamins A and E [8]. Recent studies have found that the functional compounds contained in the Sacha inchi seed oil (SIO) have nutritional and health benefits such as prevention of cardiovascular diseases, cancer, diabetes, and bacterial and viral infections, among others [9,10]. It is for this reason that its application in different industrial fields (food, cosmetics, pharmaceutical, and nutraceutical) has increased exponentially [1,9,10]. In addition, the cultivation of this plant is being implemented in social programs by governments as alternatives to coca cultivation and the economic dependence it fosters in some Amazonian regions such as Colombia [7]. This could help achieve important improvements to rural roads, access to credit, provision of formal land titles, and a reduction in violence [11]. However, the main disadvantage of oils rich in these compounds is their rapid oxidation, which results in the formation of peroxides, leading to unpleasant odors and flavors [4]. Therefore, proper control in processing, storage, and packaging is essential to preserving PUFA from oxidation [12].

Among some of the strategies to improve the stability and prolong the shelf-life of oils is the addition of antioxidants. A large number of synthetic substances—such as but16ylated hydroxyanisole (BHA), ethylenediaminetetraacetic acid (EDTA), butylated hydroxytoluene (BHT), ascorbyl palmitate, tocopherols, and gallic and ascorbic acid, among others [2,13]—have been incorporated into both oil and oil-in-water emulsions to prevent oxidation [14,15]. However, there are concerns with using synthetic antioxidants at high dosages; therefore their usage is often limited to levels below 200 ppm [2]. It has also been proposed that encapsulation with coating materials can be used to increase oil stability, reduce off-flavors, postpone lipid oxidation and enzyme hydrolysis, and allow for controlled release of encapsulated ingredients [13]. This controlled release would allow for an increase in the bioavailability of food ingredients and optimal absorption of nutrients through the body [16]. To achieve this encapsulation, an emulsion consisting of a dispersed phase of lipidic nature in a continuous phase of aqueous nature is first obtained. [17]. Food emulsions prepared using high-speed blenders, mixers, high-pressure homogenizers, microfluidizers, and ultrasound, among others, have been shown to achieve high stability against destabilization phenomena [18,19,20]. Making food emulsions using some of these techniques can be expensive on a large scale and requires considerable energy input [18]. Currently, the application of ultrasound for the manufacture of emulsions is considered a promising, effective, and environmentally friendly method compared to other high-energy emulsification methods [18,20].

To obtain the microcapsules, different drying technologies can be used, including spray-drying (SD) and freeze-drying (FD). The advantages of the SD method include ease of industrialization, cost-effectiveness, high production and throughput, compatibility with many coating materials, and the possibility of handling thermolabile materials due to the short drying times [21]. However, the quality of the spray-dried microcapsule is highly dependent on the set operating parameters [22]. On the other hand, freeze-drying is a dehydration method that produces high-quality products that are very stable and easy to handle [23]. Nevertheless, compared to other methods of product dehydration, freeze-drying is very costly due to the long processing times required [24]. Therefore, it is used in those contexts where the sensitivity of the compounds to thermal processes makes it the only possible option, or in those where the high added value of the final product justifies the costs [24,25]. In this sense, microencapsulation by combining emulsification and water-removal techniques is an interesting alternative that can be used to produce high quality microcapsules as a strategy to valorize oils with functional compounds. For example, Fidelis et al. [26] obtained freeze-dried SIO microcapsules by combining emulsification and ionic gelation techniques with sodium alginate for food fortification and to promote their protection against lipid oxidation. Da Silva Soares et al. [27] obtained SIO microcapsules with complex coacervation of ovalbumin (OVA) and sodium alginate (AL) and by applying freeze-drying. These authors evaluated the omega-3 content after in vitro gastric simulation. The results showed that the encapsulating agents used were able to protect the bioactive compounds and improve the thermal behavior of the microcapsules. Sanchez-Reinoso and Gutiérrez [28] evaluated the oxidative stability of SIO microencapsulated by spray-drying using modified starch (Hi-Cap 100) and maltodextrin. The yield and efficiency results obtained in that study indicated that SIO microcapsules are a good source of omega-3 and omega-6 fatty acids for the industry. However, to our knowledge, few studies have been conducted in which this oil is encapsulated by applying different homogenization methods and different water-removal methods. It is for these reasons that this research aims to microencapsulate and characterize SIO emulsions using two homogenization technologies: conventional homogenization (CH) and ultrasound probe homogenization (US). In addition, in this work, the influence of two drying methods, spray-drying (SD) and freeze-drying (FD), on the physicochemical properties, microstructure, and controlled release system of the resulting sacha inchi oil microcapsules were investigated. The foregoing is performed in order to provide a better understanding of the selection of the techniques that could be applied towards the manufacture of high-quality microcapsules as a strategy for the valorization of Amazonian fruits rich in oils with functional compounds.

## 2. Materials and Methods

### 2.1. Materials

The SIO used in this study was purchased from Sachacol SAS (Bucaramanga, Colombia) with 100% purity. The fatty acid composition obtained by gas chromatography analysis was 3.65% palmitic acid (C16:0), 2.48% methyl stearate acid (C18:0), 7.44% methyl oleate (Omega-9, C18:1), 34.32% methyl linoleate (Omega-6, C18:2), 51.45% methyl linolenate (Omega-3, C18:3), with an Omega-3:Omega-6 ratio of 1.5:1. The maltodextrin and sodium caseinate used in different proportions as wall materials for the emulsions were obtained from LIHUA (Funing, China) and Food System (IPF Ingredientes y Productos Funcionales S.A.S, Itagüí, Colombia), respectively. The surfactants Tween 20 and Span 80 were purchased from Croda (Campinas, Brazil). For the microcapsule characterization, the ammonium carbonate, hexane, hydrochloric acid, pepsin, and pancreatin were purchased from PanReac (ITW Reagents, Darmstadt, Germany), the sodium chloride and calcium chloride from Sigma-Aldrich (Merck, St. Louis, MO, USA), the potassium chloride from Supelco (Merck, St. Louis, MO, USA),the potassium dihydrogen phosphate from Scharlau (Scharlab, Barcelona, Spain), the sodium bicarbonate from Chemi (Bogotá, Colombia), the magnesium Chloride 6-hydrate from J.T.Baker (Fisher Scientific Madrid, Spain), and the alpha-amylase from ChemCruz (Santa Cruz Biotechnology, Dallas, TX, USA).

### 2.2. Preparation of Sacha Inchi Seed Oil Emulsions

For the preparation of the emulsions, SIO, sodium caseinate, maltodextrin, Tween 20, Span 80, and distilled water were used as ingredients. The emulsions were of the O/W (oil/water) type. A 2:1 ratio of wall material to sacha inchi oil was adjusted. The amounts of Tween and Span 80 were set at 1% and 0.5% *w*/*w*, respectively, as according to Rodriguez and Hernández-Carrión [7]. The emulsions were adjusted with distilled water to achieve 100% *w*/*w*. The process of elaboration of the emulsions was carried out in two steps, pre-homogenization and homogenization. The first step was carried out in the same way for the two homogenization technologies. The selected wall materials were dissolved separately in distilled water under constant agitation on a heating plate (Heidolph, Schwabach, Germany) at 25 °C for 30 min and then mixed with Tween 20. In the second step, the solutions of wall material with Tween 20 and the oil that had previously been mixed with Span 80 were mixed using a Quick Chef hand blender (K-MLIM50N01, Kalley, Bogotá, Colombia) in the case of conventional homogenization. For emulsions obtained by ultrasound probe homogenization, the Sonics Materials VCX 750 ultrasonic microprocessor (Sonics & Materials, Newtown, CT, USA) with 35% amplitude, 750 W, and with a pulse of 20 s on and 40 s off was used. For the two homogenization technologies, the effect of the concentration of sacha inchi seed oil (5 and 10%, *w*/*w*) and the effect of the maltodextrin:sodium caseinate ratio (90:10, 85:15, and 80:20% *w*/*w*) were studied. In addition, the effect of homogenization speed (20 and turbo) was studied in the case of emulsions prepared by conventional homogenization and the effect of time (15 and 30 min) was studied for those obtained by ultrasound probe homogenization (Table S1). The above is performed to select the proportions of the ingredients and the homogenization technology and conditions that allow for greater stability of the emulsions for subsequent drying.

### 2.3. Characterization of Sacha Inchi Seed Oil Emulsions

For all the variables under study, the analyses were performed in triplicate.

#### 2.3.1. Droplet Size

The droplet size of the emulsions was determined by using a Mastersizer 3000 particle size analyzer (Malvern Panalytical, Grovewood, United Kingdom) composed of an optical bench and a wet dispersion unit (Hydro EV) that allowed the analysis to be carried out by diluting the sample [7].

#### 2.3.2. Emulsion Stability

The stability of the emulsions was determined using a Turbiscan Lab optical analyzer (Formulaction, Worthington, OH, USA) at a temperature of 20 °C for 30 min [29]. Likewise, the creaming indices of the emulsions were determined after 7 days of storage at ambient temperature (20 °C) and refrigeration (4 °C). For this purpose, the freshly prepared emulsions were transferred to Falcon tubes and stored at ambient temperature (20 °C) or refrigerated (4 °C) for 7 days. On day 7, the creaming index was determined according to Equation (1).

IC(%) = (HS/HE)·100(1)

where HS is the height of the separated phase and HE is the initial height of the emulsion [30].

#### 2.3.3. pH and Conductivity

The pH and conductivity of the emulsions were determined using a SevenMulti multiparameter (Mettler Toledo, Columbus, OH, USA) at 20 °C.

#### 2.3.4. Rheology

The flow behavior of the emulsions was studied using an ARG2 rotational rheometer (TA Instruments, New Castle, DE, USA) using standard Peltier conical concentric cylinder geometry (997616) at ambient temperature (20 °C) and with a gap of 5920 µm. Flow behavior was studied using a shear rate between 1 and 100 s^−1^ [7]. Apparent viscosity values were reported at a shear rate of 50 s^−1^. The obtained curves were fitted to the Ostwald de Waele power law using Equation (2) for obtaining the flow index (*n*) and consistency index (*k*).

µ = *k*·γ^*n*−1^ (2)

where μ is the viscosity, *k* is the consistency index, γ is the shear rate, and *n* is the flow index.

#### 2.3.5. Interfacial Tension

The interfacial tension (IT) of the emulsions was determined using an optical tensiometer Theta Lite Attention (Biolin Scientific, Manchester, United Kingdom) with the pendant drop method [7].

### 2.4. Obtention of Nano-Structured and Macro-Structured Microcapsules

The emulsions with the best results for each homogenization technology obtained in the previous section were subjected to a spray-drying and freeze-drying process. A Mini Spray B-290 (Büchi, Flawil, Switzerland) was used for spray-drying. The drying experiments were carried out at inlet drying air temperatures of 150 °C, outlet air temperatures of 120 °C, nominal suction rates of 90% (35 m^3^/h), and nominal peristaltic pumping rates of 10% (3 mL/min). On the other hand, for freeze-drying, samples were first frozen at −80°C and then introduced into a FreeZone 6 freeze dryer (Labconco, Kansas, MO, USA). The condenser temperature and vacuum were set to −40 °C and 0.160 mbar, respectively. The dried product was collected after 96 h of processing.

### 2.5. Characterization of Nano- and Macro-Structured Microcapsules

#### 2.5.1. Moisture and Water Activity

Moisture content of SD and FD microcapsules was determined at 105 °C using a moisture analyzer (Precisa Gravimetrics, Dietikon, Switzerland). The results were expressed as a percentage of a wet basis. Water activity (a_w_) of SD and FD microcapsules was measured using a HygroPalm water activity analyzer-HP23-AW-A (Rotronic, Bassersdorf, Switzerland).

#### 2.5.2. Bulk Density

Bulk density was determined according to the methodology described by Goula and Adamopoulos [31] with modifications. Two g of SD and FD powders were transferred to a 10 mL test tube. The mass of the powder was divided by the volume occupied in the test tube to determine the bulk density.

#### 2.5.3. Solubility

The solubility or dissolution rate was determined according to the methodology described by Goula and Adamopoulos [31]. The solubility of the microcapsules was measured by adding 2 g of the material to 50 mL of distilled water at 26 °C. The mixture was stirred in a 100 mL glass beaker with a magnetic stirrer (Heidolph, Schwabach, Germany) at 900 rpm. The time required for the material to dissolve completely was recorded.

#### 2.5.4. Hygroscopicity

The hygroscopicity of the microcapsules was determined following the methodology described by Caparino et al. [32] with modifications. One g of SD and FD microcapsules each was placed and spread uniformly on a pre-weighed glass petri dish. The microcapsules and 100 mL saturated solution of sodium chloride (70% humidity) were placed inside a desiccator and left for 7 days at ambient temperature. On day 7 the glass petri dish with the samples was reweighed and, according to Equation (3), hygroscopicity of the microcapsules was determined.

HG = [(Wf − Wi)/Wi] × 100(3)

where Wf and Wi are the initial and final weight of the sample.

#### 2.5.5. Encapsulation Yield and Encapsulation Efficiency

Encapsulation yield was calculated following the methodology described by Tontul and Topuz [33] according to Equation (4). The encapsulation efficiency (EE) was calculated following the methodology described by El-Messery et al. [5] and using Equation (5):

EY(%) = (Solid matter obtained after the dehydration (g)/Weight of total solids in the feed (g))·100(4)

EE(%) = (The amount of encapsulated oil (g)/the initial oil in the formulation (g))·100(5)

#### 2.5.6. Thermogravimetric Analysis

The thermal stability of the microcapsules was evaluated by thermogravimetric analysis (TGA) using an SDTQ600 (TA Instruments, New Castle, DE, USA) following the methodology use by Amaya-Cano et al. [34]. The thermograms of the pure components, maltodextrin, and sodium caseinate were taken from the study performed by Amaya-Cano et al. [34]. The analysis of the oil component was performed to corroborate the presence of the active component (sacha inchi seed oil) in the microcapsules.

#### 2.5.7. Morphology

The morphological characteristics of the microcapsules were analyzed by scanning electron microscopy (SEM) using a Phenom ProX Desktop SEM (ThermoFisher, Waltham, MA, USA). The samples were coated with gold nanoparticles using Desk V Thin Film Deposition Solution (Denton Vacuum, Moorestown, NJ, USA). The images were taken with the detector within the lens using an acceleration voltage of 15 kV. Likewise, the microcapsules obtained by SD were subjected to image analysis to determine the Feret diameter using ImageJ software (version 1.53).

#### 2.5.8. Bioavailability of Microcapsules through In Vitro Digestion Studies

The bioavailability of the SIO microcapsules was evaluated following the methodology described by Amaya-Cano et al. [34]. For the simulation of digestive fluids under in vitro conditions, the methodology described by Minekus et al. [35] was followed. The residence times in the simulated digestive fluids and the pH values were adjusted according to the description provided by Amaya-Cano et al. [34]. A UV/VIS Evolution 60 S spectrophotometer (Thermo Scientific, Waltham, MA, USA) was used to initially determine the wavelength where sacha inchi oil presents the highest absorbance peak. Subsequently, the absorbance of the encapsulated oil was determined at a wavelength of 230 nm (value determined in the previous test). In addition, the concentration of oil released was calculated using the Beer–Lambert law and Equation (6).

A = ε·C·L(6)

where A is the absorbance, ε is the extinction coefficient of sacha inchi seed oil = 0.48 L/mol⋅cm, C is the molar concentration, and L is the length of the quartz cell = 1 cm.

### 2.6. Statistical Analysis

For each homogenization technology in the encapsulation process, a 2^2^ × 3 factorial design was carried out. For both homogenization technologies, the effect of the concentration of sacha inchi seed oil (5 and 10%, *w*/*w*) and the effect of the maltodextrin:sodium caseinate ratio (90:10, 85:15, and 80:20%, *w*/*w*) were studied. In addition, the effect of homogenization speed (20 and turbo) was studied in the case of emulsions prepared by conventional homogenization, and the effect of time (15 and 30 min) for those obtained by the ultrasound probe homogenization (Table S1). For the emulsions subjected to the drying process, a 2^2^ factorial design was performed. The effect of the drying technology (spray-drying and freeze-drying) and the effect of the homogenization technology (conventional and ultrasound probe) were studied (Table S1). Descriptive statistics were performed by calculating the mean and standard deviation for each response variable under study. The data were subjected to an analysis of variance (ANOVA) using the Minitab^®^ statistical program (version 18.1) with a significance level of 5% using Tukey’s mean comparison test.

## 3. Results

### 3.1. Characterization of Sacha Inchi Seed Oil Emulsions

Table 1 and Table 2 show the results of the characterization of the sacha inchi seed oil emulsions obtained by conventional and ultrasound homogenization, respectively.

#### 3.1.1. Droplet Size

The droplet size is defined as the diameter of the internal phase droplet, which is of vital importance for an emulsion since it influences the texture and stability of the emulsion, associating a uniform and small particle size with an emulsion with good stability [7,36]. The droplet sizes were expressed in volume D(4,3). Values for emulsions made by conventional homogenization ranged from 0.77 to 2.21 μm (Table 1) and 0.31 to 1.19 μm for those obtained by ultrasound probe homogenization (Table 2). The fabricated CH emulsion showed the highest D(4,3) values, while the D(4,3) values of US treatment were the lowest. These results could be due to the cavitation effect of ultrasound treatment, which is responsible for the decrease in the emulsion droplet size. In addition, this decrease in droplet size may also be due to the fact that US treatment is a high-energy method compared to CH and, therefore, more efficient in decreasing emulsion droplet size [20].

Based on the statistical results, it is concluded that all the factors and their interactions had a significant effect on the droplet size, where the factor that had the greatest influence was the velocity for CH emulsions and the time for US emulsions. The values of D(4,3) show that, as the velocity of the homogenization treatment increased, the particle size decreased, with the smallest particle sizes obtained for emulsions prepared using a turbo velocity. This finding coincides with that reported by Ricaurte et al. [37], where the oil particle size was affected by the homogenization treatment velocity. In addition, if the sonication time or power is increased, the physical forces of acoustic cavitation become stronger, which aids in the formation of smaller oil droplets and the promotion of emulsion modifiers [20].

It was also found that, as the oil concentration increased for both homogenization methods, the droplet size increased. This phenomenon is related to the fact that a higher oil concentration increases the viscosity of the emulsion, which contributes to increasing the resistance to the process speed and, therefore, the recoalescence between the oil droplets. On the other hand, when comparing the values of the runs where the weight percentage of sodium caseinate was higher, it was also found that the droplet size was smaller. Amaya-Cano et al. [34] investigated the encapsulation of chia seed oil by varying different proportions of wall material (maltodextrin and sodium caseinate), finding that, in those formulations where the amount of caseinate was higher, the particle size was reduced, indicating that the protein limits the size of the oil particles.

#### 3.1.2. Stability Index

Emulsions are thermodynamically unstable when the interfacial area between the two phases is large. This characteristic is common in oil-in-water emulsions due to the large interfacial area between the water phase and the oil phase [37]. The tendency to reorganize towards their initial state leads to local changes in droplets’ size and concentration. Therefore, the stability analysis is based on the measurement of these changes from the backscatter readings and the calculation of the stability index (TSI), indicating that the higher the TSI value, the more unstable the emulsion [38].

The results obtained for the physical stability (TSI) of the emulsions obtained with conventional and ultrasound probe homogenization during a 30 min period can be seen in Table 1 and Table 2. The physical stability values for freshly prepared emulsions obtained by CH ranged between 1.63 and 2.99 (Table 1). Likewise, the physical stability values for freshly prepared emulsions obtained by US ranged between 2.13 and 4.28 (Table 2). Based on these results it could be observed that for both homogenization technologies the emulsions with the highest stability were those made with a lower oil concentration (5% *w*/*w*), 80:20 formulation of wall material (maltodextrin: sodium caseinate), higher speed (conventional homogenization), and 30 min of homogenization (ultrasound probe).

When performing the statistical analysis with a significance level of 5% it was found that, for both technologies, the factor that has the greatest influence on the stability of the emulsions was the maltodextrin:sodium caseinate ratio. This may be related that at higher concentrations of protein, interfaces can form on the particle surface; these layers can be extensive enough to dull the attractive van der Waals forces, resulting in repulsion between the droplets and further stabilization of the emulsion [39]. Furthermore, it was observed that speed, homogenization time, and oil concentration contribute to the physical stabilization of emulsions. This result indicates that as the oil concentration increases, the emulsion stability decreases. This may be due to the formation of aggregates resulting from the increased collision between the oil droplets [15].

For the creaming index, emulsions were observed 7 days after preparation. Table 1 and Table 2 show that emulsions prepared with a 10% *w*/*w* of oil concentration were the most unstable, with higher oil phase separation at the top of the Falcon tube. In addition, the emulsions stored at ambient temperature for both homogenization technologies shows a higher creaming index in comparison to emulsions stored at 4 °C. This may be due to the effect of temperature, which causes the droplets to approach each other, increasing their size and rate of coalescence [19,40].

#### 3.1.3. pH and Conductivity

The results of the pH study for the emulsified systems at different process conditions are shown in Table 1 and Table 2. The results of the pH study were very similar to each other, with values between 6.51 and 6.68 for the emulsions obtained by conventional homogenization and 6.53 to 6.68 for the emulsions obtained by the ultrasound probe.

The pH of the emulsions must be taken into account to prevent the emulsions from reaching the point of minimum solubility. This effect causes precipitation of proteins, which prevents the continuation of the drying procedures to which the emulsions can be subjected. For an emulsion to be stable, the pH value of the emulsion must be greater than the isoelectric point of the protein (sodium caseinate), i.e., 5.98 [41].

Based on the ANOVA results, it was established that the factors with the greatest influence on the two homogenization technologies were the wall material ratio and the oil concentration. The emulsions with higher sodium caseinate concentration (15 and 20% *w*/*w*) in the wall material ratio and higher oil concentration (10% *w*/*w*) reported higher pH values. In different studies, it was found that for pH close to or below the isoelectric point, changes in the electrostatic and hydrophobic interactions of proteins are promoted, which can lead to the formation of insoluble agglomerates and, therefore, a destabilization of the emulsions [42]. On the other hand, since the pH of the emulsions was higher than the isoelectric point of the mentioned sodium caseinate, it can be said that the emulsions were stable.

The results of conductivity assays for the emulsified systems with different process conditions are shown in Table 1 and Table 2. For the emulsion obtained by CH, the values ranged between 685 and 816 μS/cm, and the values for the emulsions obtained by US ranged between 683 and 831 μS/cm. For the two homogenization technologies, all the factors and their interactions affect the conductivity, but the factor that had the greatest influence on the conductivity was the oil concentration (*p* < 0.005). These results coincide with those reported in the literature where it is established that when the content of the aqueous phase exceeds that of the oily phase, the conductivity of an emulsion increases [19,37]. In this sense, it is logical that higher values are reported for those emulsions with lower oil concentrations (5% *w*/*w*).

Likewise, the observed increase in conductivity could also be related to the increase in the proportion of sodium caseinate. This is due to the ability of this protein to prevent agglomeration, because it can act as an emulsifier due to its amphiphilic nature, increasing the repulsion of oil and water particles [19,43].

#### 3.1.4. Rheology

The characteristics of an emulsion are strongly influenced by its rheological properties. Some of them such as the consistency coefficient (k), the apparent viscosity, and the flow index (*n*) are remarkable rheological properties for describing the behavior of an emulsion [44]. Studies on these properties are useful and important for food applications, processing, quality control, and sensory evaluation [45]. These properties depend largely on the emulsion phases (continuous and dispersed), but mainly on the dispersed phase. This not only changes the emulsion viscosity with respect to the viscosity of the phases but also makes the emulsion behavior different from the simplest case, the Newtonian fluid [26].

**Figure 1:**
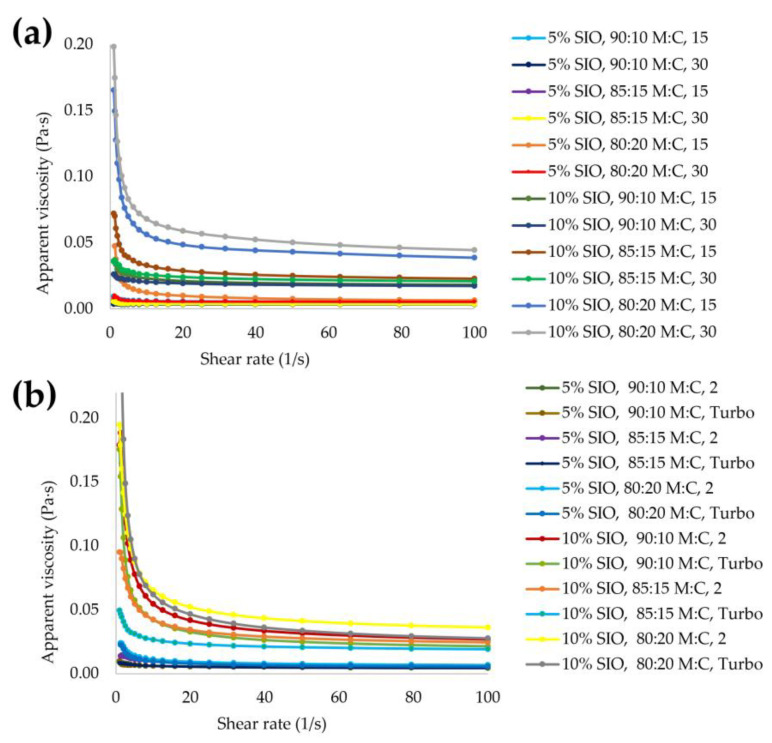
(**a**) Flow behavior of sacha inchi seed oil emulsions made with conventional homogenization and (**b**) ultrasound probe homogenization.

The apparent viscosity of different emulsions as a function of shear rate are shown in Figure 1. From this figure, it can be affirmed that the emulsions present a pseudoplastic behavior, characteristic of those fluids that decrease their viscosity as the applied shear rate increases [45]. The viscosity values at a shear rate of 50 s^−1^ varied within the range of 0.004 and 0.040 Pa·s for CH (Table 1) and 0.005 and 0.042 Pa·s for US (Table 2). It was found that the emulsions that presented higher viscosity for the two technologies were those elaborated with a higher concentration of oil (10% *w*/*w*) and a higher proportion of wall material (80:20 *w*/*w*).

When the statistical analysis was performed, all the factors and interactions were significant; however, the one that had the greatest influence was oil concentration, which reaffirms that the highest viscosity values are those of the emulsions made with a 10% *w*/*w* concentration. This coincides with the findings reported by McClements [19], who found that the greater the aggregation of oil droplets, the higher the viscosity of the emulsion. This same rheological behavior was observed in the elaboration of SIO emulsions with sodium alginate developed by Silva et al. [26]. In that study, when the incorporation of SIO in the emulsion was increased (75%), there was an increase in the viscosity of the emulsions. On the other hand, it was also evidenced that increasing the amount of caseinate in the wall material ratio increased the viscosity. This may be due to the fact that the higher the protein concentration, the higher the emulsifying capacity [46]. In addition, it could be observed that the group of emulsions elaborated with the US (Figure 1b) presented lower values compared to the emulsions obtained by CH (Figure 1a). This coincides with a study reported by Zhou et al. [47] wherein the emulsions elaborated with ultrasound-assisted emulsification reported the lowest values compared to the emulsions obtained by high-pressure homogenization and high-speed homogenization. Likewise, Kaltsa et al. [48] prepared oil-in-water emulsions with olive oil and whey protein concentrate (WPC) using high-intensity ultrasound. In that study, it was found that the application of ultrasound decreased the apparent viscosity of all emulsions.

According to Zhou et al. [20], the Taylor equation (Equation (7)) can describe how the viscosity decrease is related to the application of ultrasound. This equation describes how the droplet size or radius (r) is directly proportional to the interfacial tension (τ) and inversely proportional to the viscosity of the continuous phase (µ). For that reason, it makes sense that the emulsions obtained by the US treatment present a decrease in apparent viscosity compared to CH, since the droplet size is lower than the emulsions obtained by conventional homogenization.

r ~ τ /µγ(7)

The values of n and k obtained from the Ostwald–de Waele power law are shown in Table 3. The coefficients of determination R^2^ were greater than 0.90, which indicates that this model has a good fit. Since n < 1 we can state that all emulsions are non-Newtonian pseudoplastic fluids [49]. In addition, the emulsions subjected to US reported higher n values and lower k values, which indicate that the emulsions have higher fluidity under external forces [20]. The results obtained in this study agree with those reported by Qayum et al. [50]. They used ultrasound to prepare an emulsion of soybean oil and α-lactalbumin, finding that application of US increased the values of n from 0.528 to 0.595 while the values of k decreased from 0.0164 Pa⋅s to 0.0111 Pa⋅s.

#### 3.1.5. Interfacial Tension

The interfacial tension (IT) for the emulsions prepared by CH (Table 1) varied across a range of values from 33.54 to 37.91 (mN/m) and the ST of the emulsions obtained by US (Table 2) varied with a range of values from 33.91 to 38.34 (mN/m). The ANOVA statistical analysis of the emulsions showed that for the two homogenization technologies, the oil concentration and the maltodextrin:sodium caseinate ratio were the factors that had the greatest influence on interfacial tension. It was observed that increasing the concentration of sodium caseinate (15 and 20%) in the wall material ratio decreased the surface tension at the interface, which may be due to the action of the protein in decreasing the free energy, decreasing the interfacial area, and—in turn—generating more thermodynamic stability in the system [51]. In previous studies, a reduction in the stress on the air–water interface was found with increasing protein concentrations, indicating that, when using the protein at 0.1 g/L, tension values of around 54.2 mN/m were reached, while the tension values were reduced to around 42.2 mN/m when the concentration was increased to 3 g/L [52]. This coincides with the results obtained in the present study, where emulsions made with a caseinate concentration of 10% for the wall material ratio reported values between 37.77 and 40.51 mN/m for emulsions obtained by CH (Table 1) and between 37.58 and 39.40 for emulsions obtained by US (Table 2). In addition, while working with sodium caseinate concentrations of 15 and 20% in the wall material ratio, values between 33.54 and 37.39 mN/m were reported for emulsions obtained by CH (Table 1) and between 33.91 and 36.46 mN/m for emulsions obtained by the US (Table 2).

### 3.2. Characterization of Nano- and Macro-Structured Microcapsules

Based on the results obtained in the previous section, it was established that, for both homogenization technologies, the emulsion with the best results in terms of higher stability, lower creaming index, lower droplet size, lower interfacial tension, and higher conductivity was the one with an oil concentration of 5% *w*/*w*, a wall material ratio of 80:20 (maltodextrin:sodium caseinate), and, finally, a turbo homogenization speed in the case of CH and a time of 30 min for the emulsion obtained by US. Table 4 shows the experimental design for obtaining and characterizing SIO microcapsules by conventional and ultrasound probe homogenization subjected to SD and FD.

#### 3.2.1. Moisture and Water Activity

The shelf-life and quality of the dried microcapsules depend mainly on moisture content, water activity, preparation/storage temperature, design of the dryer, physical and chemical properties of the feed, and the operating parameters of the process [53,54]. According to Klinkesorn et al. [55], to guarantee the shelf-life of dried microcapsules in the food industry, the maximum moisture content is 3–4%. The results showed that the moisture content of the microcapsules obtained by SD and FD were below these specifications, which indicates that they were microcapsules with a longer shelf-life. However, it is important to note that spray-drying yielded lower moisture content (1.64–1.67%) compared to freeze-drying (1.74–1.76%) (Table 4). This coincides with that reported by Pellicer et al. [53] and Quispe-Condori et al. [54], where lower values were obtained for samples subjected to spray-drying compared to freeze-drying. It has also been found that lower moisture values in SD may be due to the effect of higher temperatures in the process. 

The a_w_ values for SD and FD microcapsules ranged between 0.07–0.11% and 0.03–0.09, respectively, and the drying methods were significantly different from each other (*p* < 0.05). The water activity values of the microcapsules for both drying technologies were below the maximum specification of 0.2 for spray-dried industrial powders [56] and within the recommended limit to guarantee stable dry powders (<0.30) [57,58]. The lower a_w_ values for FD microcapsules can be attributed to the effect of the high vacuum in this process. The lower amount of a_w_ in the microcapsules obtained by FD may be due to the influence of the vacuum gradient on the water transfer rate. Moayyedi et al. [59] obtained the lowest a_w_ values for freeze-dried samples (0.052–0.072) compared to electrospray (0.15) and spray-drying methods (0.12–0.15).

#### 3.2.2. Bulk Density 

The bulk densities of spray-dried and freeze-dried microcapsules varied between 0.42 and 0.45 g/cm^3^ and between 0.20 and 0.22 g/cm^3^, respectively. The results show that the spray-dried microcapsules present higher values compared to the freeze-dried ones (*p* < 0.05). This may be due to the structural difference between spray-dried and freeze-dried microcapsules. According to Franceschinis et al. [60] during FD the freezing conditions determine the size of the generated crystals, allowing them to maintain their structure, and as a result, the pore size distribution also remained. Additionally, it is possible that the high temperature employed in spray-drying degraded the wall material, creating a more compact and rigid product. This is in agreement with the results reported by Caliskan and Dirim [61], where the bulk densities of freeze-dried sumac extract powders (0.267–0.282 g/mL) were significantly lower than those of spray-dried sumac extract powders (0.369–0.508 g/mL).

#### 3.2.3. Solubility

Solubility rates of the spray-dried and freeze-dried microcapsules ranged between 65.50 and 73.50 s and 24.65 and 26.55 s, respectively. These results show that the solubility rate for freeze-dried microcapsules is considerably better than that of spray-dried microcapsules (*p* < 0.05). The high solubility times of the microcapsules obtained by SD may be associated with the tendency of the products subjected to this process to aggregate. According to Liu et al. [62], aggregation may affect the particle dissolution rate. Likewise, it has been reported that the particle size of the samples could influence the solubility time since the smaller the particles, the higher their surface energy, which causes the particles to stick to each other and, thus, have less contact with water [63]. In this sense, it is logical that microcapsules obtained by SD with small and regular shapes present longer solubility times compared to the irregular particles typical of freeze-dried microcapsules. These results also agree with those reported by Karthik and Anandharamakrishnan [64], where the solubility rate for the capsules obtained by SD ranged from 89 to 102 s while the capsules obtained by FD ranged from 36 to 48 s.

#### 3.2.4. Hygroscopicity

The hygroscopicity values varied from 2.32 to 5.07%. These results show that the hygroscopicity in US for both drying technologies is considerably better than that of CH (*p* < 0.05). This may be due to the hygroscopicity of the microcapsules generally being related to their composition, type, the concentration of the carriers, and the size of the microcapsules [33]. Nurhadi et al. [65] state that microcapsules with values of more than 20% are considered very hygroscopic. Different studies have found that a high hygroscopicity produces stickiness in the powder, which contributes to a decrease in shelf-stability. In this study, the values were less than 20%, indicating a low hygroscopicity for the microcapsules, thus facilitating their shelf-life and stability during storage.

#### 3.2.5. Encapsulation Yield and Encapsulation Efficiency

The encapsulation yield of microcapsules is presented in Table 4. Lower encapsulation yields were obtained with spray-drying (53.33–57.72%) than with freeze-drying (82.79–86.04%). This may be because the high temperatures increased the stickiness of the microcapsules by directly degrading the wall materials, causing less dry matter to be recovered. This coincides with what was reported by Quispe-Condori et al. [54], where lower particle yields were obtained by SD than FD using the same ratio of zein:linseed oil. Since the calculated encapsulation yield values were greater than 50%, it can be concluded that the drying process was successful [33].

The highest EE was obtained by SD with values of 69.90 to 70.18%, followed by FD with values of 60.02 to 60.16%. The same behavior was observed in the study conducted by Anwar and Kunz [66], where higher EE values were obtained for SD (83.62%) than FD (less than 50%). Additionally, El-Messery et al. [5] reported results showing that spray-drying provides a higher encapsulation efficiency than freeze-drying, obtaining values between 62.2 and 78.8% for SD microcapsules and between 51.9 and 58.2% for FD microcapsules. The results of the present study coincide with those reported by Amaya-Cano et al. [34], where the same coating materials (maltodextrin:sodium caseinate) were used to encapsulate chia oil by spray-drying. In that study the encapsulation efficiencies were higher than 65% and increased as the amount of sodium caseinate increased.

#### 3.2.6. Thermogravimetric Analysis

**Figure 2:**
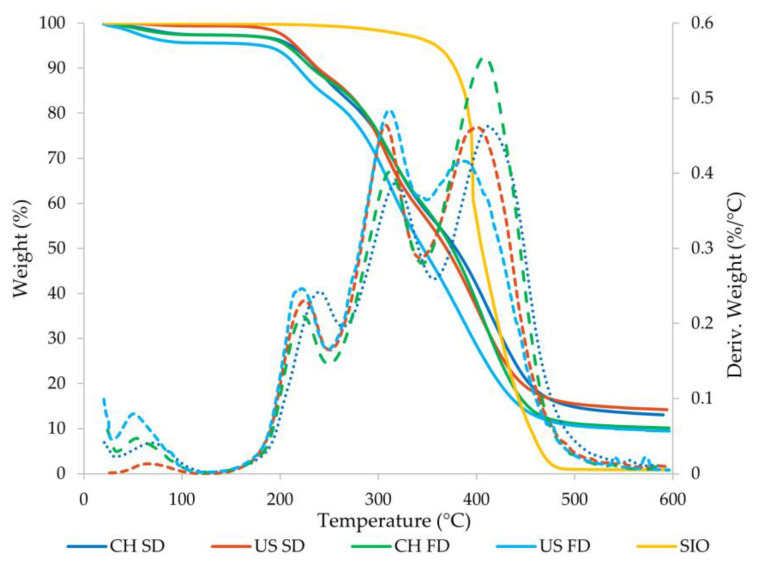
TGA of sacha inchi seed oil microcapsules prepared with CH and US and dried by SD and FD. (—) Weight loss. (---) Derived from weight loss.

For each of the samples prepared with different homogenization methods and different drying methods, the same behavior was observed (Figure 2). Weight losses occurred in three common temperature ranges as shown in Figure 2. The first range corresponds to temperatures between 45 °C and 110 °C. This weight loss is related to the evaporation of water remaining in the sample or perhaps resulting from rehydration of the microcapsule during storage [34,67]. The second weight loss corresponded to degradation of the wall materials, which was observed between 200 and 350 °C. This coincides with that reported in the literature, where the degradation of maltodextrin and sodium caseinate was found to occur at temperatures of approximately 300 °C [34,68,69]. The latter range corresponds to temperatures between 350 and 490 °C, corresponding to the volatilization of the sacha inchi seed oil fraction. As shown in the figure, at temperatures above 500 °C there is no significant variation in the percentage of weight lost, which is an indication that there is no residual oil. This coincides with the findings reported by Amaya-Cano et al. [34], who observed that, at temperatures above 500 °C, no variations in weight were perceived, indicating that there were no chia seed oil remnants. In Figure 2, it is possible to observe that the microcapsules obtained by SD presented a better EE than the microcapsules obtained by FD, since the microcapsules presented a greater amount of residue when the curves reach the maximum temperature, which is associated with a greater amount managing to overcome degradation by temperature. Thermal analysis of both drying technologies showed that microcapsules have great potential for evaluation in food processing applications. This is because they showed considerable thermal stability, as there is no significant mass loss at the usual temperatures (100–250 °C) of processes such as baking, frying, sterilization, pasteurization, and ultra-pasteurization, among others.

#### 3.2.7. Morphology

**Figure 3:**
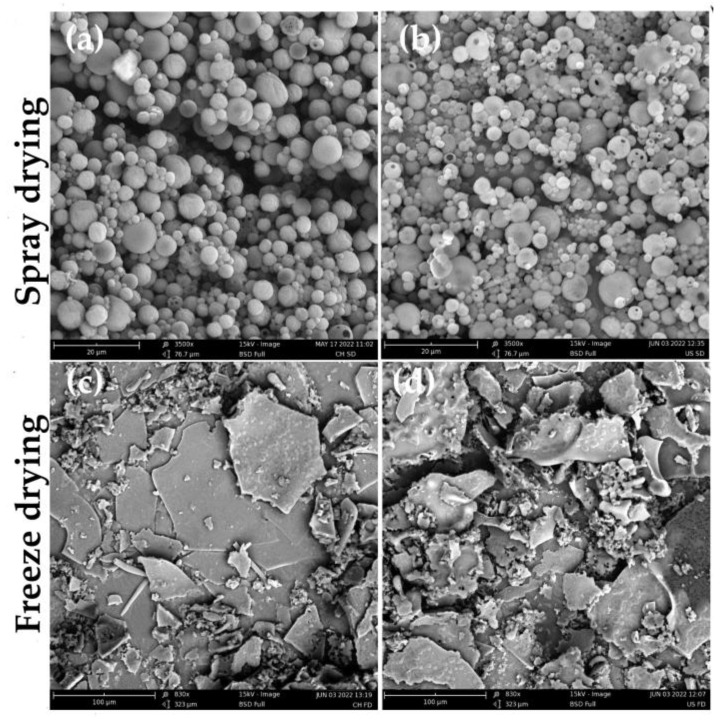
Scanning electron microscopy images. (**a**) Conventional homogenization spray-drying, (**b**) Ultrasound probe homogenization spray-drying, (**c**) Conventional homogenization freeze-drying, (**d**) Ultrasound probe homogenization freeze-drying.

The morphology analysis of the microcapsules from the experiments for SD and FD is shown in Figure 3. When the images were compared, it became clear that the particle morphology showed a marked difference between encapsulated microcapsules. Figure 3a,b shows that the microcapsules exhibited various sizes and regular and spherical shapes, characteristic of microcapsules obtained by spray-drying. Additionally, in these images, it can be seen that some of the microcapsules obtained by SD present a few cracks and rough surfaces. These characteristics can be attributed to the rapid evaporation of the water and the rapid cooling to which the samples are subjected during the drying process [58]. On the other hand, the morphology of FD microcapsules (Figure 3c,d) exhibited an irregular shape with some pores on the surface. These morphological characteristics in the microcapsules obtained by FD are because, during the freezing stage, ice crystals are formed and then sublimate, which allows them to maintain their structure and, consequently, their pore size distribution [70]. These results agree with those reported by Caparino et al. [32], where mango microcapsules subjected to different drying methods presented the same structure as those obtained in this study.

Several studies [71,72] have found that microcapsules obtained by SD exhibit higher EE compared to FD. According to El-Messery et al. [5], this is because the regular and spherical shapes of the microcapsules obtained in the spray-drying process contribute to a decrease in the permeability of the encapsulates to gases and provides extra protection to and retention of the core material.

In the image analysis it was found that the Feret diameter of the microcapsules subjected to SD was larger for those obtained by CH (4.75 ± 2.194 μm) compared to those obtained by US (3.11 ± 0.991 μm). This reduction in particle size is due to the cavitation effect of ultrasound. Additionally, these results agree with those reported by Wang et al. [73]. In that study, ultrasound was applied for the preparation of hemp seed oil microcapsules with soy protein isolate and it was found that, with increasing ultrasound power, particle size decreased and was relatively uniform.

#### 3.2.8. Capsule Release into Simulated Human Digestive Fluids

Lipid digestion occurs in the small intestine, mainly due to the presence of pancreatic lipase [74]. According to Teng et al. [75], the presence of bioactive compounds at the end of intestinal digestion is a key factor in determining whether they can be effectively utilized by humans. For this reason, the amount of alpha-linolenic acid (omega-3) at the end of intestinal digestion was evaluated. The omega-3 concentrations found by means of the Beer–Lambert law are shown in Table 4. These results show that for both homogenization technologies, the highest concentrations were found in the samples subjected to SD. This is because the samples dried by SD presented a higher EE than the FD samples and, therefore, the amount of oil released in the SIF phase was higher. El-Messery et al. [5] found that samples with higher EE at 8 and 10% krill oil obtained by SD also presented higher bioaccessibility values. In addition, it was found that microcapsules obtained with CH presented lower values of omega-3 concentrations. This could be related to the hygroscopicity of these samples (higher values). This parameter is of vital importance for oil encapsulates, since the presence of water can cause stickiness in the microcapsules and lead to lipid oxidation. On the other hand, studies have found that a reduction in particle size can increase the bioavailability of micronutrients and phytocomponents [36]. In this sense, it is consistent that the microcapsules obtained by US presented the highest values of omega-3 concentration. Da Silva Soares et al. [27] investigated microparticles containing SIO subjected to gastric conditions to investigate the efficacy of microencapsulation using ovalbumin and sodium alginate. The results found in that study indicate that the microcapsule protected the acyl omega-3 units in comparison to when the free SIO was subjected to the same gastric conditions. This strengthens the claim that microencapsulation with coating materials is an important alternative for protecting functional compounds such as PUFA.

## 4. Conclusions

In this work it was possible to evaluate the influence of different combinations of wall materials, SIO concentrations, and different homogenization technologies. By analyzing the results, it was possible to establish a criterion to recommend a formulation for both homogenization technologies that favors the stability of the emulsions. These formulations are those in which the oil concentration is 5% *w*/*w* and the maltodextrin:sodium caseinate ratio is 80:20 *w*/*w*; the speed with which the best results were obtained was turbo, in the case of conventional homogenization, and a time of 30 min for the ultrasound probe. The effect of two different drying approaches was also evaluated with respect to their physicochemical properties, encapsulation stability, and in vitro bioavailability. The results showed that both spray- and freeze-drying are regarded as efficient tools to design controlled release delivery systems. However, due to its greater scalability and lower operating costs, spray-drying proved more versatile than freeze-drying. Finally, the results of the present study can provide a new perspective on drying technologies and open the door to the advantages of generating emulsions for the encapsulation of valuable oils to protect various food components. Additionally, encapsulates containing SIO show high potential, which makes them interesting for applications in foods to enrich them with the fatty acids contained in this oil—components that are essential for metabolism and contribute to the prevention of diseases.

## Figures and Tables

**Table 1:** Experimental design for preparation of emulsions with conventional homogenization and physicochemical properties evaluated: droplet size, stability index, creaming index (4 and 20 °C), pH, conductivity, viscosity, and interfacial tension (IT).

Concentration SIO (%*w*/*w*)	Maltodextrin: Sodium Caseinate (%*w*/*w*)	Velocity	Droplet Size (μm)	Stability Index (TSI)	Creaming Index 20 °C	Creaming Index 4 °C	pH	Conductivity (μS/cm)	Viscosity at a Shear Rate of 50 s^−1^ (Pa·s)	IT (mN/m)
5	90:10	20	1.88 ^c^(0.014)	2.20 ^b^(0.016)	71.43	28.57	6.55 ^d^(0.004)	695 ^ef^(0.707)	0.005 ^fg^(<0.01)	37.77 ^ab^(0.191)
5	90:10	Turbo	1.67 ^d^(0.028)	2.16 ^bc^(0.028)	65.71	20.00	6.51 ^e^(0.005)	706 ^def^(5.657)	0.005 ^fg^(<0.01)	37.64 ^ab^(0.113)
5	85:15	20	1.30 ^f^(0.014)	1.86 ^d^(0.044)	64.29	8.57	6.66 ^ab^(0.005)	816 ^a^(8.485)	0.006 ^g^(<0.01)	36.46 ^bcd^(0.184)
5	85:15	Turbo	1.05 ^g^(0.042)	1.88 ^d^(0.006)	57.14	0.00	6.66 ^ab^(0.009)	802 ^ab^(4.243)	0.005 ^g^(<0.01)	35.07 ^ef^(0.085)
5	80:20	20	0.92 ^h^(0.045)	1.65 ^e^(0.072)	17.14	0.00	6.67 ^a^(0.005)	805 ^ab^(5.657)	0.008 ^e^(<0.01)	34.46 ^efg^(0.474)
5	80:20	Turbo	0.77 ^i^(0.004)	1.63 ^e^(0.040)	8.57	0.00	6.64 ^bc^(0.001)	794 ^b^(6.364)	0.008 ^e^(<0.01)	35.52 ^cde^(0.297)
10	90:10	20	2.21 ^a^(0.021)	2.99 ^a^(0.013)	84.29	42.86	6.54 ^d^(0.005)	685 ^f^(7.071)	0.033 ^ab^(0.002)	37.91 ^a^(0.092)
10	90:10	Turbo	2.07 ^b^(0.064)	2.80 ^a^(0.108)	78.57	37.14	6.53 ^de^(0.012)	689 ^ef^(2.828)	0.025 ^bcd^(<0.01)	36.78 ^abc^(0.014)
10	85:15	20	1.46 ^e^(0.035)	1.97 ^cd^(0.054)	37.14	11.43	6.64 ^c^(0.001)	708 ^de^(2.828)	0.027 ^cd^(0.001)	35.15 ^de^(0.926)
10	85:15	Turbo	0.92 ^h^(0.004)	2.02 ^bcd^(0.032)	28.57	0.00	6.63 ^c^(0.006)	702 ^def^(2.121)	0.021 ^d^(<0.01)	35.49 ^cde^(0.092)
10	80:20	20	0.79 ^i^(0.004)	1.94 ^d^(0.018)	28.57	14.29	6.68 ^a^(0.004)	738 ^c^(4.950)	0.042 ^a^(0.001)	33.74 ^fg^(0.064)
10	80:20	Turbo	0.85 ^hi^(0.002)	2.05 ^bcd^(0.071)	20.00	4.29	6.67 ^a^(0.002)	718 ^cd^(9.192)	0.028 ^bc^(0.001)	33.54 ^g^(0.361)

For the same column, different letters indicate the presence of statistically significant differences according to Tukey’s test (*p* < 0.05). Values in parentheses are standard deviations.

**Table 2:** Experimental design for preparation of emulsions with an ultrasound probe and physicochemical properties evaluated: droplet size, stability index, pH, conductivity, viscosity, and interfacial tension (IT).

Concentration SIO (%*w*/*w*)	Maltodextrin: Sodium Caseinate (%*w*/*w*)	Time	Droplet Size (μm)	Stability Index (TSI)	Creaming Index 20 °C	Creaming Index 4 °C	pH	Conductivity (μS/cm)	Viscosity at a Shear Rate of 50 s^−1^ (Pa·s)	IT (mN/m)
5	90:10	15	0.98 ^c^(0.003)	3.41 ^c^(0.117)	75.71	60.00	6.56 ^f^(0.010)	796.50 ^bc^(9.192)	0.005 ^de^(0.001)	37.08 ^ab^(1.273)
5	90:10	30	0.47 ^g^(0.006)	2.80 ^ef^(0.104)	29.43	11.43	6.68 ^a^(0.001)	727.50 ^d^(4.950)	0.040 ^a^(0.004)	33.91 ^c^(0.651)
5	85:15	15	0.33 ^i^(0.007)	2.55 ^fg^(0.067)	20.00	14.29	6.56 ^f^(0.129)	717.50 ^de^(4.950)	0.038 ^ab^(0.004)	34.00 ^c^(0.778)
5	85:15	30	0.31 ^ij^(0.004)	2.13 ^hi^(0.051)	7.14	0.00	6.66 ^ab^(0.008)	831.50 ^a^(4.950)	0.008 ^d^(0.000)	35.20 ^bc^(0.318)
5	80:20	15	0.50 ^g^(0.001)	3.20 ^cd^(0.032)	17.43	7.14	6.64 ^bcd^(0.066)	816.00 ^ab^(2.828)	0.005 ^de^(0.001)	37.33 ^ab^(0.410)
5	80:20	30	0.38 ^h^(0.003)	2.28 ^gh^(0.002)	7.14	0.00	6.61 ^cde^(0.062)	802.00 ^bc^(4.243)	0.004 ^e^(0.001)	37.39 ^ab^(0.806)
10	90:10	15	1.08 ^b^(0.021)	3.83 ^b^(0.078)	74.43	48.57	6.53 ^g^(0.006)	683.00 ^g^(5.657)	0.019 ^c^(0.001)	38.09 ^a^(0.113)
10	90:10	30	0.76 ^d^(0.011)	2.63 ^f^(0.070)	11.43	12.86	6.64 ^bcd^(0.003)	700.00 ^efg^(7.071)	0.026 ^abc^(0.002)	35.67 ^bc(^0.127)
10	85:15	15	0.28 ^j^(0.004)	1.95 ^i^(0.106)	8.29	0.00	6.65 ^abc^(0.002)	789.00 ^c^(9.899)	0.005 ^de^(0.001)	36.35 ^ab^(0.537)
10	85:15	30	0.89 ^d^(0.002)	2.90 ^de^(0.092)	58.57	41.43	6.61 ^cde^(0.005)	712.50 ^def^(3.536)	0.004 ^e^(0.001)	37.58 ^a(^0.566)
10	80:20	15	0.67 ^f^(0.010)	2.83 ^ef^(0.032)	8.57	2.86	6.62 ^cde^(0.013)	706.50 ^def^(4.950)	0.025 ^abc^(0.005)	36.09 ^abc^(1.280)
10	80:20	30	1.19 ^a^(0.014)	4.28 ^a^(0.164)	65.71	60.00	6.54 ^fg^(0.012)	689.00 ^fg^(1.414)	0.023 ^bc^(0.005)	38.34 ^a^(0.474)

For the same column, different letters indicate the presence of statistically significant differences according to Tukey’s test (*p* < 0.05). Values in parentheses are standard deviations.

**Table 3:** Flow index (*n*), consistency index (k), and R^2^ for sacha inchi seed oil emulsions prepared with conventional and ultrasound probe homogenization.

SIO (%*w*/*w*)	Maltodextrin: Sodium Caseinate (%*w*/*w*)	Homogenization Technology	Velocity/Time	*n*	*k* Pa·s^n^	R^2^
5	90:10	CH	2	0.850	0.129	0.966
5	90:10	CH	Turbo	0.876	0.124	0.970
5	85:15	CH	2	0.820	0.162	0.975
5	85:15	CH	Turbo	0.857	0.126	0.995
5	80:20	CH	2	0.699	0.199	0.975
5	80:20	CH	Turbo	0.679	0.191	0.986
10	90:10	CH	2	0.707	0.344	0.973
10	90:10	CH	Turbo	0.649	0.364	0.968
10	85:15	CH	2	0.679	0.361	0.982
10	85:15	CH	Turbo	0.793	0.261	0.968
10	80:20	CH	2	0.625	0.467	0.973
10	80:20	CH	Turbo	0.458	0.559	0.949
5	90:10	US	15	0.900	0.121	0.923
5	90:10	US	30	0.965	0.088	0.967
5	85:15	US	15	0.930	0.165	0.930
5	85:15	US	30	0.916	0.160	0.945
5	80:20	US	15	0.568	0.244	0.952
5	80:20	US	30	0.882	0.124	0.938
10	90:10	US	15	0.847	0.230	0.958
10	90:10	US	30	0.914	0.202	0.976
10	85:15	US	15	0.754	0.300	0.946
10	85:15	US	30	0.874	0.235	0.972
10	80:20	US	15	0.606	0.439	0.966
10	80:20	US	30	0.427	0.519	0.954

**Table 4:** Experimental design for SD and FD microcapsules and physicochemical properties evaluated: moisture, a_w_, solubility, density, hygroscopicity, encapsulation yield (EY), encapsulation efficiency (EE), and concentration of omega-3.

Homogenization Technology	Drying Technology	Moisture (%)	Water Activity (a_w_)	Bulk Density (g/cm^3^)	Solubility (s)	Hygroscopicity (g/100 g)	EY (%)	EE (%)	[Omega-3] (M)
CH	SD	1.67 ^b^(0.007)	0.11 ^a^(0.001)	0.42 ^b^(0.021)	65.50 ^b^(0.707)	5.07 ^a^(0.006)	53.33	69.90	1.68
US	SD	1.64 ^b^(0.021)	0.07 ^c^(0.005)	0.45 ^b^(0.006)	73.50 ^a^(2.121)	2.32 ^b^(0.038)	57.72	70.18	1.75
CH	FD	1.76 ^a^(0.021)	0.09 ^b^(0.001)	0.20 ^a^(0.004)	24.65 ^c^(1.626)	4.56 ^a^(0.243)	82.79	60.02	1.60
US	FD	1.74 ^a^(0.007)	0.03 ^d^(0.002)	0.22 ^a^(0.001)	26.55 ^c^(0.276)	2.37 ^b^(0.156)	86.04	60.16	1.63

For the same column, different letters indicate the presence of statistically significant differences according to Tukey’s test (*p* < 0.05).

## Data Availability

Data is contained within the article or supplementary material.

## Supplementary Materials

The following supporting information can be downloaded at: https://www.mdpi.com/article/10.3390/foods11243950/s1, Table S1: Experimental design for the preparation of SIO emulsions and experimental design for the obtention of nano and macro-structured microcapsules.
