# Supplementary material for: Obtention of Sacha Inchi (Plukenetia volubilis Linneo) Seed Oil Microcapsules as a Strategy for the Valorization of Amazonian Fruits: Physicochemical, Morphological, and Controlled Release Characterization
Source: Foods. 2022 Dec 7;11(24):3950. doi: 10.3390/foods11243950 (PMC9777982; doi:10.3390/foods11243950)
Supplement: Supplementary file 1 [file foods-11-03950-s001.zip › foods-2070975-supplementary.pdf]

Table SM1. Experimental design for the preparation of SIO emulsions and experimental design for the obtention of nano and macro-structured microcapsules.

	Homogenization technology	Factors	Levels
Preparation of SIO emulsions	Conventional	%SIO(*w*/*w*)	5, 10
		Maltodextrine:Sodium caseinate (%, *w*/*w*)	90:10, 85:15, 80:20
		Speed	20, turbo
	Ultrasound probe	%SIO(*w*/*w*)	5, 10
		Maltodextrine:Sodium caseinate (%, *w*/*w*)	90:10, 85:15, 80:20
		Time (min)	15, 30
Obtention of nano and macro-structured microcapsules	Spray drying	Homogenization technology	Conventional, ultrasound
	Freeze drying	Homogenization technology	Conventional, ultrasound
